# Threshold-dominated regulation hides genetic variation in gene expression networks

**DOI:** 10.1186/1752-0509-1-57

**Published:** 2007-12-06

**Authors:** Arne B Gjuvsland, Erik Plahte, Stig W Omholt

**Affiliations:** 1 Department of Animal Science and Aquaculture, Norwegian University of Life Sciences, 1432 Ås, Norway; 2 Department of Chemistry, Biotechnology and Food Science, Norwegian University of Life Sciences, 1432 Ås, Norway; 3 Centre for Integrative Genetics (CIGENE), Norwegian University of Life Sciences, 1432 Ås, Norway

## Abstract

**Background:**

In dynamical models with feedback and sigmoidal response functions, some or all variables have thresholds around which they regulate themselves or other variables. A mathematical analysis has shown that when the dose-response functions approach binary or on/off responses, any variable with an equilibrium value close to one of its thresholds is very robust to parameter perturbations of a homeostatic state. We denote this threshold robustness. To check the empirical relevance of this phenomenon with response function steepnesses ranging from a near on/off response down to Michaelis-Menten conditions, we have performed a simulation study to investigate the degree of threshold robustness in models for a three-gene system with one downstream gene, using several logical input gates, but excluding models with positive feedback to avoid multistationarity. Varying parameter values representing functional genetic variation, we have analysed the coefficient of variation (*CV*) of the gene product concentrations in the stable state for the regulating genes in absolute terms and compared to the *CV *for the unregulating downstream gene. The sigmoidal or binary dose-response functions in these models can be considered as phenomenological models of the aggregated effects on protein or mRNA expression rates of all cellular reactions involved in gene expression.

**Results:**

For all the models, threshold robustness increases with increasing response steepness. The *CV*s of the regulating genes are significantly smaller than for the unregulating gene, in particular for steep responses. The effect becomes less prominent as steepnesses approach Michaelis-Menten conditions. If the parameter perturbation shifts the equilibrium value too far away from threshold, the gene product is no longer an effective regulator and robustness is lost. Threshold robustness arises when a variable is an active regulator around its threshold, and this function is maintained by the feedback loop that the regulator necessarily takes part in and also is regulated by. In the present study all feedback loops are negative, and our results suggest that threshold robustness is maintained by negative feedback which necessarily exists in the homeostatic state.

**Conclusion:**

Threshold robustness of a variable can be seen as its ability to maintain an active regulation around its threshold in a homeostatic state despite external perturbations. The feedback loop that the system necessarily possesses in this state, ensures that the robust variable is itself regulated and kept close to its threshold. Our results suggest that threshold regulation is a generic phenomenon in feedback-regulated networks with sigmoidal response functions, at least when there is no positive feedback. Threshold robustness in gene regulatory networks illustrates that hidden genetic variation can be explained by systemic properties of the genotype-phenotype map.

## Background

### Historical perspective

In the early 1970s, Leon Glass, Stuart Kauffman, and René Thomas started their pioneering efforts in exploring the possibility of modelling what was then called "Genetic Control Circuits" (Thomas) and "Biochemical Control Networks" (Glass and Kauffman) by using concepts and ideas from mathematical logic. Combining these ideas with earlier ideas from Monod and others on allostery and cooperativity which suggested sigmoidal rate dependences of key metabolites, Glass and Kauffman [1,2] and Thomas [3] proposed that gene transcription could be modelled by sigmoidal response functions depending on transcription factor concentrations. In the case of several transcription factors acting on a gene, they assumed the effect could be expressed by Boolean combinations of the separate response functions, and proposed a simple framework of ordinary differential equations for modelling of gene regulatory networks based on these principles. Glass and Kauffman observed that the behaviour of these regulatory networks was remarkably insensitive to the steepness of the sigmoids, and suggested to use Heaviside or step function in stead of sigmoids as dose-response functions to simplify the models and their analysis.

From these early attempts, phenomenologic frameworks for the modelling of Gene Regulatory Networks (GRNs) have been developed, based on a few fundamental premises: (i) genes are controlled by transcription factors (TFs) which combine into logical input functions, and these can be described by Boolean logic; (ii) the effect of a transcription factor on the transcription rate of a gene (the response function) can be described by a sigmoidal function of its concentration with a pronounced threshold behaviour (graded response) or by a Heaviside step function (binary response); (iii) this can be modelled in a discrete way in which transcription factors are either absent of present, and proteins are either transcribed or not, or in a continuous way by means of ordinary differential equations; (iv) proteins act as transcription factors, so that networks become closed with feedback loops; (v) posttranscriptional, translational and posttranslational regulation, transport processes, metabolic processes etc. can be phenomenologically encompassed by the sigmoidal or binary response functions.

Recent experimental evidence seem to confirm the validity and usefulness of the basic assumption on which GRNs are resting. A number of studies have shown that gene networks have *cis*-regulatory elements governed by a Boolean-like logic [4-11]. There is also extensive experimental documentation of sigmoidal or binary responses in gene regulation [5,11-16] as well as theoretical justifications based on classical principles from physical chemistry [17-20].

How common is steep transcription response? Analyses based on classical methods from statistical physical chemistry show that a steep transcription response curve could be due to cooperativity in the transcription factor binding [18-20]. It has been shown that transcriptional and signalling cascades do in fact lead to graded or binary responses [21,22]. There is also extensive evidence that transcription response in single cells is binary (see references in [14]), and that individual cells responds in an on/off way to varying external inputs [13]. Thus, there are good reasons to expect that high gain regulation is quite common in gene regulatory networks.

The method developed in [23-27] to deal with models with steep sigmoidal response functions works for quite general models, also with other nonlinearities in addition to the steep sigmoidal functions. In the course of this work it was discovered that when the responses functions are very steep, equilibrium values for actively regulating variables show a remarkable robustness towards changes in all parameters except the level of the threshold around which the active regulation occurs. We call this phenomenon *threshold robustness*. To be precise, this is a mathematical result valid in the limit when the sigmoid function approaches a step function (Heaviside function), but for continuity reason it is also valid for steep sigmoid functions. But to what extent is it found in models with more empirically sound threshold functions? We have investigated this question by a simulation study of a wide class of 3-dimensional regulatory systems where the regulatory dose-response relationships are varied from a hyperbolic Michaelis-Menten situation to an extremely steep sigmoidal situation.

If conserved when the steepness of the sigmoidal interactions is slackened to realistic values, insensitivity or robustness to functional genetic polymorphism may be a deep generic property of some of the loci in a wide range of regulatory networks. When present, threshold robustness adds significant and characteristic phenomena to the genotype-phenotype map. This implies for example that the functional mutational changes in network which shows threshold robustness will only results in small phenotypic variations in the homeostatic values of the protein products.

### Analytical foundation

The above basic assumptions of Gene Regulatory Networks lead to the following generic model for the time course of gene product concentrations:

y˙j=κjRj(Z)−λjQj(Z)yj,
 MathType@MTEF@5@5@+=feaafiart1ev1aaatCvAUfKttLearuWrP9MDH5MBPbIqV92AaeXatLxBI9gBaebbnrfifHhDYfgasaacPC6xNi=xI8qiVKYPFjYdHaVhbbf9v8qqaqFr0xc9vqFj0dXdbba91qpepeI8k8fiI+fsY=rqGqVepae9pg0db9vqaiVgFr0xfr=xfr=xc9adbaqaaeGacaGaaiaabeqaaeqabiWaaaGcbaGafmyEaKNbaiaadaWgaaWcbaGaemOAaOgabeaakiabg2da9GGaciab=P7aRnaaBaaaleaacqWGQbGAaeqaaOGaemOuai1aaSbaaSqaaiabdQgaQbqabaGccqGGOaakcqWGAbGwcqGGPaqkcqGHsislcqWF7oaBdaWgaaWcbaGaemOAaOgabeaakiabdgfarnaaBaaaleaacqWGQbGAaeqaaOGaeiikaGIaemOwaOLaeiykaKIaemyEaK3aaSbaaSqaaiabdQgaQbqabaGccqGGSaalaaa@4703@

where *y*_*j *_is the concentration of gene product number *j*, *j *= 1,...,*n*, *Z*_*j *_= *S*(*y*_*j*_, *θ*_*j*_, *p*_*j*_) is a sigmoid or binary function with threshold *θ*_*j *_and steepness parameter *p*_*j*_, and *y *and *Z *are the vectors with *y*_*j *_and *Z*_*j *_as components. The functions *R*_*j *_∈ [0, 1] and *Q*_*j *_∈ ⟨0, 1] are regulatory functions, frequently taken to be algebraic equivalents of Boolean functions [25], describing the regulation of production and decay, respectively, while the positive parameters *κ*_*j *_and *λ*_*j *_represent the maximal production and decay rates.

Eq. (1) could be justified in at least two ways. It could be considered a model of transcription regulation with the *y*_*j *_still representing protein concentrations. This model could be derived from a larger model for protein and mRNA concentrations where transcription of mRNA is regulated by protein concentrations, and the conversion from mRNA to protein is described by linear equations. If all mRNA degradation rates are much larger than all protein degradation rates, we can apply a quasi-stationary hypothesis to the mRNA concentrations, leading to Eq. (1). This procedure can be justified mathematically as well as biologically. A simple example is presented below, and the case *n *= 2 is studied in [28].

Alternatively, taken as a model of gene regulation, Eq. (1) is a generic phenomenological model of protein concentration dynamics, not a mechanistic description of gene regulation. The threshold functions model the aggregated effect of all the processes involved in the real cellular regulatory networks [29]: transcription, translation, intracellular transport, post-translational modifications, protein-protein interactions, metabolic processes, and signalling cascades. Such drastic simplification is hard to justify theoretically, but models based on the generic Eq. (1) have been applied successfully to many real systems [5,30,31]. Considered in this way, Eq. (1) is a generic, phenomenological framework assumed to catch the essential features of a wide range of regulatory systems, where the regulatory control may be at the level of transcription, mRNA stability, translation, or post-translation, and where the state variables may for example be concentrations of proteins, hormones, mRNA, and intracellular ions [29].

**Figure 1:**
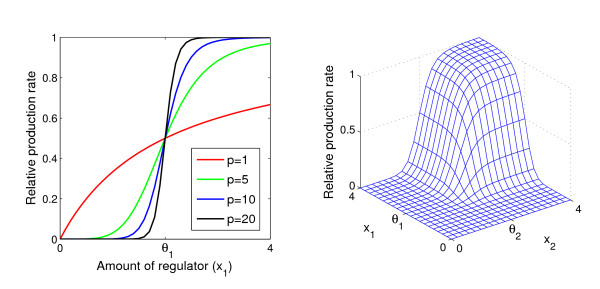
Regulatory functions used in the simulations. (a) The Hill function describes the dose-responce relationship between the amount of a regulator *x*_1 _and the relative production rate of the regulated gene. The threshold parameter *θ*_1 _= 2 is the same for all four curves. (b) Regulatory function for two regulators *x*_1 _and *x*_2_. Two Hill functions are combined by the algebraic equivalent of the Boolean AND function (see Table 1). The parameter values used in this panel are *θ*_1 _= *θ*_2 _= 2 and *p *= 10.

In almost all cases, regulation of the degradation is disregarded, thus we assume *Q*_*j *_= 1. We let *S *be a Hill function, *Z*_*j *_= *S*(*y*_*j*_, *θ*_*j*_, *p*) = *y*_*j*_^*p*^/(*y*_*j*_^*p *^+ *θ*_*j*_^*p*^), with the property that when *p *→ ∞, then *Z*_*j *_approaches the Heaviside step function with threshold *θ*_*j*_, and put all *p*_*j *_= *p *(Fig. 1). Of course, in real systems sigmoids most likely do not have the same steepness. Our justification for taking all *p*_*j *_equal is to investigate systematically whether there is a threshold robustness effect for varying steepnesses. Once this effect is established, one might take one step further to investigate necessary and sufficient conditions on the steepnesses for robustness in more realistic models.

Models encompassed by Eq. (1) have been extensively investigated mathematically, in particular in the special case of *S*_*j *_being step functions. (See [32] for an extensive list of references.) An efficient way to analyse models of the generic type Eq. (1) with steep sigmoids is presented in [26,27].

Frequently models encompassed by the generic type Eq. (1) have stationary points lying close to the thresholds of one or several variables when the sigmoids are steep, i.e. when *p *is large. A stationary point y*(*p*) is called a singular stationary point (SSP) for Eq. (1) if at least one of its components approaches its threshold when *p *→ ∞. These components are called singular, the others regular. It has been proved that if a SSP Y* exists in the step function limit, then there exists a stationary point y*(*p*) for sufficiently large *p *with the property that y*(*p*) → Y* when *p *→ ∞ [26]. Mathematically, SSPs of Eq. (1) have remarkable generic robustness properties. The key issue is that the singular components of Y* are locked to their thresholds, while the regular components vary with the other parameters in the model. For y*(*p*) this implies that when *p *is large, the singular components are highly insensitive to changes in all parameters except the thresholds of the singular variables. Biologically this insensitivity means that the expression levels of genes corresponding to singular components will be kept virtually constant despite stochastic or mutational variation in the expression process rates.

An illustrative example is provided by a popular model for a negatively autoregulated gene, which are very common in e.g. *E. coli*

m˙=r+α(1−Z)−γm,y˙=κm−λy,
 MathType@MTEF@5@5@+=feaafiart1ev1aaatCvAUfKttLearuWrP9MDH5MBPbIqV92AaeXatLxBI9gBaebbnrfifHhDYfgasaacPC6xNi=xI8qiVKYPFjYdHaVhbbf9v8qqaqFr0xc9vqFj0dXdbba91qpepeI8k8fiI+fsY=rqGqVepae9pg0db9vqaiVgFr0xfr=xfr=xc9adbaqaaeGacaGaaiaabeqaaeqabiWaaaGcbaqbaeWabiqaaaqaaiqbd2gaTzaacaGaeyypa0JaemOCaiNaey4kaSccciGae8xSdeMaeiikaGIaeGymaeJaeyOeI0IaemOwaOLaeiykaKIaeyOeI0Iae83SdCMaemyBa0MaeiilaWcabaGafmyEaKNbaiaacqGH9aqpcqWF6oWAcqWGTbqBcqGHsislcqWF7oaBcqWG5bqEcqGGSaalaaaaaa@46C6@

where *m *and *y *are the concentrations of mRNA and protein, respectively, *Z *= *S*(*y*, *θ*, *p*) is the sigmoidal response function, *r *is the basal transcription rate, *θ *is the regulation threshold, *p *is the steepness parameter, and the remaining four parameters are production and degradation rate constants. Incidentally, asssuming quasistationarity of mRNA concentration as explained above, leads to y˙
 MathType@MTEF@5@5@+=feaafiart1ev1aaatCvAUfKttLearuWrP9MDH5MBPbIqV92AaeXatLxBI9gBaebbnrfifHhDYfgasaacPC6xNi=xH8viVGI8Gi=hEeeu0xXdbba9frFj0xb9qqpG0dXdb9aspeI8k8fiI+fsY=rqGqVepae9pg0db9vqaiVgFr0xfr=xfr=xc9adbaqaaeGacaGaaiaabeqaaeqabiWaaaGcbaGafmyEaKNbaiaaaaa@2D57@ = (*κ*/*γ*)[*r *+ *α*(1 - *Z*)] - *λy*, which is of the generic type Eq. (1).

**Figure 2:**
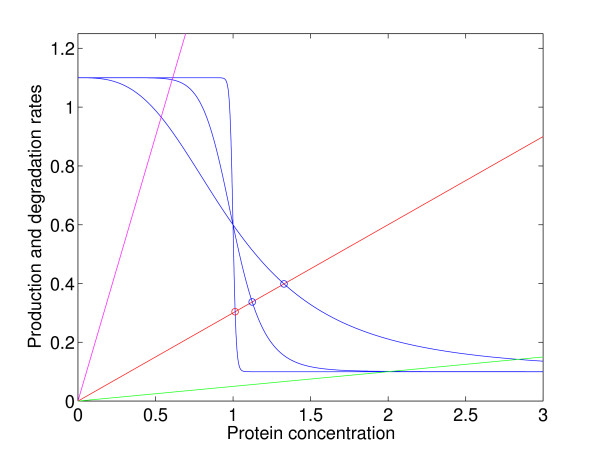
Graphical solution of the equilibrium condition of Eq. (2) for varying steepness of the response function and varying relative degradation rates. The blue curves are graphs of *r *+ *α*(1 - *Z*), the straight lines graph the degradation term *γy*. For intermediate values of *γ *the equilibrium concentration is close to *θ *and approaches *θ *when the response function steepness increases (red line), and there is active regulation. When *γ *is small, the basal transcription rate is sufficient to balance the degradation, and y* gets large (green line). When *γ *is large, degradation is so rapid that the protein concentration never reaches the level where it regulates. Maximal production is necessary to balance the degradation (magenta line).

After elimination of *m*, the equilibrium equation for *y *can be solved graphically (Fig. 2). There are three qualitatively different situations to consider: the solution can be close to the threshold and approach the threshold as steepness increases (red line), or the gene can be almost off (green line) or almost constitutively on (magenta line), the differences being accentuated for higher steepness. In the first case the stationary point is a SSP, in the two other cases it is regular. In the singular case we find the solution in the step function limit by putting y* = *θ *and solve for m*, getting m* = *λθ*/*κ*, Z* = 1 + *r*/*α *- *g*, where *g *= *γλθ*/(*ακ*). From the requirement Z* ∈ ⟨0, 1⟩ it follows that this solution exists when *r*/*α *<*g *< 1 + *r*/*α*. Thus, when *g *lies in this interval, the protein concentration y* is very close to the threshold, independent of parameter values, and y* = *θ *in the step function limit.

However, if *g *is perturbed outside this interval, y* is no longer locked to the threshold, and robustness is lost. If *p *→ ∞ and *g *<*r*/*α*, then Z* = 1 (the magenta case), m* = *r*/*γ*, y* = *κr*/(*γλ*), while if *g *> 1 + *r/α*, then Z* = 0 (the green case), m* = (*α *+ *r*)/*γ*, y* = *κ*(*α *+ *r*)/(*γλ*). We see that in both these cases, y* vary with the other parameters.

### Model system

For our simulations we chose a particular realisation of Eq. (1) which after a scaling is given by the dimensionless equations

**Figure 3:**
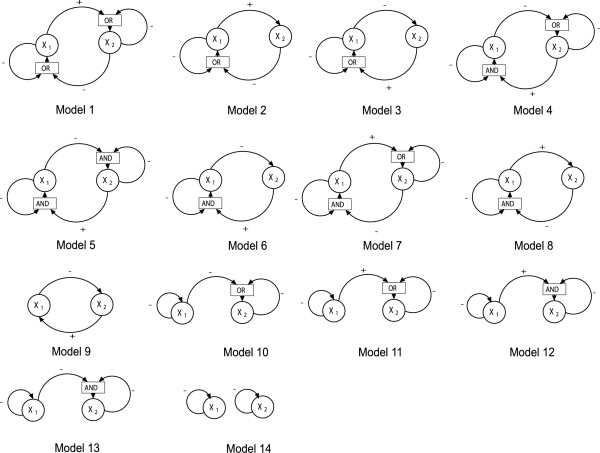
Connectivity diagrams for the 14 network models in the simulation study. Genes 1 and 2 are represented by circles, the downstream gene 3 being omitted for clarity. The sign of an arrow indicates whether the type of regulation is activation (+), in which case the input variable is *Z*_*i*_, or inhibition (-), in which case the input variable is 1 - *Z*_*i*_. When a gene has two regulators, the individual signals are combined with a logic block, represented by a rectangle, merging the two signals into one by the continuous analogue of the Boolean functions AND or OR. (See the Methods section for explanations of the Boolean variables and functions.)

*x*_*j*_*' *= *α*_*j*_*R*_*j*_(*Z*_1_, *Z*_2_) - *γ*_*j *_*x*_*j*_,   *j *= 1,...,3,

where *x*_*j *_is the scaled version of *y*_*j*_, *α*_3 _∈ ⟨0, 1⟩ and all *γ*_*j *_∈ ⟨0, 1⟩, and *Z*_*j *_= *x*_*j*_^*p*^/(*x*_*j*_^*p *^+ 1). The prime denotes differentiation with respect to a scaled time. The Boolean functions *R*_*j *_are chosen so that the system has a unique stable point in which both *x*_1 _and *x*_2 _are singular (close to threshold at equilibrium). Altogether 14 models satsify this requirement (Fig. 3, see also the Methods section).

The key question is whether the robustness of SSPs is still generic and preserved when the Hill exponents atttain smaller, more realistic values. Considering instances of Eq. (1) as models for gene regulatory networks, we checked this out for a large set of 3-dimensional particular realisations of Eq. (3). We took the set of stable equilibrium values xj∗(p)
 MathType@MTEF@5@5@+=feaafiart1ev1aaatCvAUfKttLearuWrP9MDH5MBPbIqV92AaeXatLxBI9gBaebbnrfifHhDYfgasaacPC6xNi=xH8viVGI8Gi=hEeeu0xXdbba9frFj0xb9qqpG0dXdb9aspeI8k8fiI+fsY=rqGqVepae9pg0db9vqaiVgFr0xfr=xfr=xc9adbaqaaeGacaGaaiaabeqaaeqabiWaaaGcbaGaemiEaG3aa0baaSqaaiabdQgaQbqaaiabgEHiQaaakiabcIcaOiabdchaWjabcMcaPaaa@32EA@ as the phenotype and the set {*α*_*j*_, *γ*_*j*_, *θ*_*j*_, *p*, *R*_*j*_}, *j *= 1,...,3, of parameter values and regulatory functions as the genotype. The equilibrium conditions for Eq. (3) then define the genotype-phenotype map for this system. Our interest is to investigate the robustness properties of the phenotype under mutations, i.e. under perturbations of the genotype. For the 14 models with a unique SSP in the step function limit we investigated the robustness properties of the singular and the regular components of the SSP for a range of parameter perturbations and for varying Hill exponent of the response functions from *p *= 1 (Michaelis-Menten conditions) to *p *= 100, which for all practical purposes is very close to a step function.

The coefficient of variation *CV *for a distribution is defined as the ratio of the standard deviation to the mean. Being a dimensionless number and scaled by the mean, it is suitable for comparison of the variation of distributions with large differences in mean values. To measure and compare the *CV*s for the equilibrium values xi∗
 MathType@MTEF@5@5@+=feaafiart1ev1aaatCvAUfKttLearuWrP9MDH5MBPbIqV92AaeXatLxBI9gBaebbnrfifHhDYfgasaacPC6xNi=xH8viVGI8Gi=hEeeu0xXdbba9frFj0xb9qqpG0dXdb9aspeI8k8fiI+fsY=rqGqVepae9pg0db9vqaiVgFr0xfr=xfr=xc9adbaqaaeGacaGaaiaabeqaaeqabiWaaaGcbaGaemiEaG3aa0baaSqaaiabdMgaPbqaaiabgEHiQaaaaaa@2FC3@ for each of the 14 models we generated 81 parameter sets, giving a total of 1134 different systems with a unique SSP for which *x*_1 _and *x*_2 _are singular and *x*_3 _regular. For each data set we sampled 50 random pertubations of each production parameter *α*_*j *_from the uniform distribution *U*(*α*_*j*_/2, 3*α*_*j*_/2) with corresponding coefficient of variation *CV*_uni _≈ 0.288. We then computed the coefficients of variation *CV*_*j*_^*k*^, *j *= 1, 2, 3, *k *= 1,...,81 for the steady state levels of all three variables in all the 14 networks separately. Details are described in the Methods section. We use the minimum coefficients of variation as robustness measure in order to be able to compare the robustness of most favourable parameter sets.

## Results

**Figure 4:**
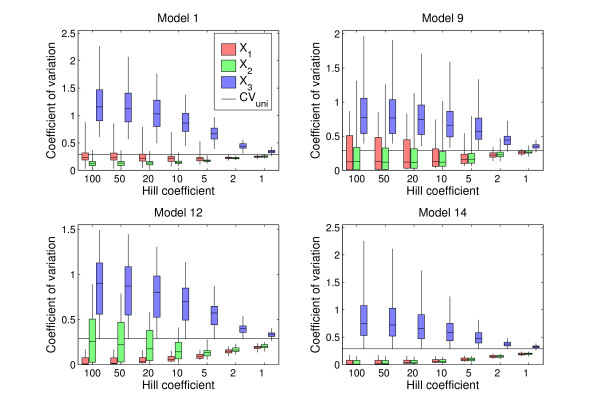
Variation in steady state values for Models 1, 9, 12, and 14. Boxplots show the distributions of the 81 coefficients of variance for all three genes using seven different Hill coefficients ranging from *p *= 1 to *p *= 100. For each Hill coefficients the three plots show from left to right the coefficient of variation for gene 1 (red), 2 (green), and 3 (blue), respectively. The boxes show the quartiles and the median. The black vertical lines extend to the largest observation and the smallest. The long black horisontal line shows the coefficient of variation 0.288 of the perturbed production rates *α*_*j*_.

Models 1, 9, 12, and 14 represent four different classes among the 14 models: Model 1 represents models with a negative feedback loop between the two genes plus autoregulation, Model 9 has just a pure negative feedback loop and no autoregulation, Model 12 has interaction but no feedback loop between genes 1 and 2, and Model 14 has no interaction at all between genes 1 and 2 (Fig. 3). The comparison (Fig. 4) of the distributions of *CV*_1_^*k *^and *CV*_2_^*k *^over the 81 parameter sets to the distribution of *CV*_3_^*k *^shows that there is a marked difference between the CVs of the singular variables *x*_1 _and *x*_2 _and the downstream, regular variable *x*_3_. This difference is most marked for high Hill coefficient, but is present even under Michaelis-Menten conditions. While in almost all cases the variation in x3∗
 MathType@MTEF@5@5@+=feaafiart1ev1aaatCvAUfKttLearuWrP9MDH5MBPbIqV92AaeXatLxBI9gBaebbnrfifHhDYfgasaacPC6xNi=xH8viVGI8Gi=hEeeu0xXdbba9frFj0xb9qqpG0dXdb9aspeI8k8fiI+fsY=rqGqVepae9pg0db9vqaiVgFr0xfr=xfr=xc9adbaqaaeGacaGaaiaabeqaaeqabiWaaaGcbaGaemiEaG3aa0baaSqaaiabiodaZaqaaiabgEHiQaaaaaa@2F5C@ is larger than *CV*_uni_, the variations in x1∗
 MathType@MTEF@5@5@+=feaafiart1ev1aaatCvAUfKttLearuWrP9MDH5MBPbIqV92AaeXatLxBI9gBaebbnrfifHhDYfgasaacPC6xNi=xH8viVGI8Gi=hEeeu0xXdbba9frFj0xb9qqpG0dXdb9aspeI8k8fiI+fsY=rqGqVepae9pg0db9vqaiVgFr0xfr=xfr=xc9adbaqaaeGacaGaaiaabeqaaeqabiWaaaGcbaGaemiEaG3aa0baaSqaaiabigdaXaqaaiabgEHiQaaaaaa@2F58@ and x2∗
 MathType@MTEF@5@5@+=feaafiart1ev1aaatCvAUfKttLearuWrP9MDH5MBPbIqV92AaeXatLxBI9gBaebbnrfifHhDYfgasaacPC6xNi=xH8viVGI8Gi=hEeeu0xXdbba9frFj0xb9qqpG0dXdb9aspeI8k8fiI+fsY=rqGqVepae9pg0db9vqaiVgFr0xfr=xfr=xc9adbaqaaeGacaGaaiaabeqaaeqabiWaaaGcbaGaemiEaG3aa0baaSqaaiabikdaYaqaaiabgEHiQaaaaaa@2F5A@ are considerably smaller for most parameter sets, in Model 14 for all parameter sets. The result for Model 14 is not surprising, as it is well known that negative autoregulation leads to a high degree of robustness [30,33,34].

What is more important is that in almost all cases, all the coefficients of variation for genes 1 and 2 are significantly smaller than those for gene 3. The reduced sensitivity of gene 3 when *p *decreases towards 1 can be seen as a consequence of the fact that when the sigmoids in the rate equation for *x*_3 _are slackened, a certain variation in the inputs gives smaller variation in the response. However, for all the models except Model 14 there are parameter sets for which the system is not robust, which shows that robustness is generally only present for a certain range of parameter values. The decisive factor is how easily a perturbation shifts the position of the stable point away from the switching domain. If this happens, the gene's status in the regulatory system is changed: it is no longer an active regulator and its robustness is lost. Accordingly, the gene that was regulated is now either off or constitutively on unless it is still effectively regulated by the other gene.

**Figure 5:**
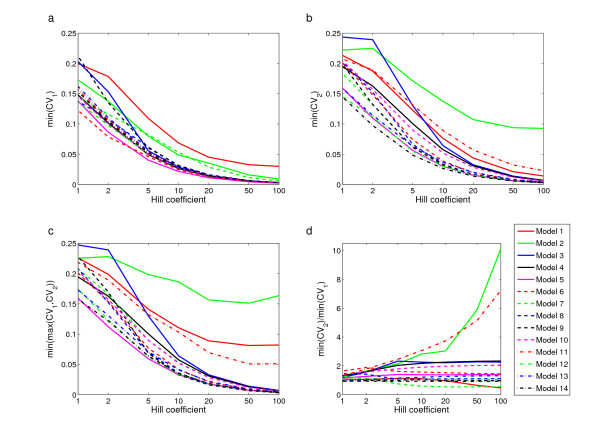
The coefficient of variation as function of the Hill coefficient for the most robust parameter sets for gene 1 and 2 across all 14 models. (a)-(b): Minimum of *CV*_1_^*k *^and *CV*_2_^*k*^, respectively, over all 81 parameter sets. (c): *CV*_minmax _as function of the Hill coefficient for each of the 14 models. An explanation for the remarkably high values for Models 1, 2, and 11 for high Hill coefficient values is given in the text. (d) The ratio of minimum of *CV*_2_^*k *^to minimum of *CV*_1_^*k *^as function of the Hill coefficient for each of the 14 models.

For all 14 models a decrease in *p *reduces robustness of both singular variables, defined as the minima of *CV*_1_^*k *^and *CV*_2_^*k *^over all 81 parameter sets (Fig. 5). But even at *p *= 1 there is less variation in the equilibrium values than in the perturbed parameter values. The cases of highest robustness for each of the 14 models and all steepness values show large variation among the models (Fig. 5). But for all models, even for the least robust Models 1 and 2, robustness increases with increasing Hill exponent, and is always smaller than *CV*_uni_. In Fig. 5c the differences among the models are accentuated for high Hill exponents. For each model the number *CV*_minmax _plotted along the vertical axis is computed as follows: for each of the 81 parameter sets first compute CVmax⁡k
 MathType@MTEF@5@5@+=feaafiart1ev1aaatCvAUfKttLearuWrP9MDH5MBPbIqV92AaeXatLxBI9gBaebbnrfifHhDYfgasaacPC6xNi=xH8viVGI8Gi=hEeeu0xXdbba9frFj0xb9qqpG0dXdb9aspeI8k8fiI+fsY=rqGqVepae9pg0db9vqaiVgFr0xfr=xfr=xc9adbaqaaeGacaGaaiaabeqaaeqabiWaaaGcbaGaem4qamKaemOvay1aa0baaSqaaiGbc2gaTjabcggaHjabcIha4bqaaiabdUgaRbaaaaa@33C9@ = max{*CV*_1_^*k*^, *CV*_2_^*k*^}, then find *CV*_minmax _= min{CVmax⁡k
 MathType@MTEF@5@5@+=feaafiart1ev1aaatCvAUfKttLearuWrP9MDH5MBPbIqV92AaeXatLxBI9gBaebbnrfifHhDYfgasaacPC6xNi=xH8viVGI8Gi=hEeeu0xXdbba9frFj0xb9qqpG0dXdb9aspeI8k8fiI+fsY=rqGqVepae9pg0db9vqaiVgFr0xfr=xfr=xc9adbaqaaeGacaGaaiaabeqaaeqabiWaaaGcbaGaem4qamKaemOvay1aa0baaSqaaiGbc2gaTjabcggaHjabcIha4bqaaiabdUgaRbaaaaa@33C9@}. For each model this procedure selects the parameter set for which both *CV*_1 _and *CV*_2 _are small, giving *CV*_minmax _as a measure of the highest robustness when both genes 1 and 2 are taken into account.

**Figure 6:**
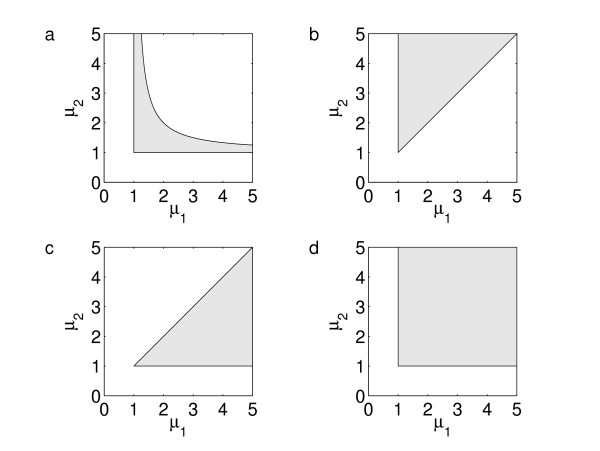
The shaded areas are the robustness domains Ω_SSP _in the (*μ*_1_, *μ*_2_)-plane for (a) Models 1, 2, 11, (b) Models 3, 13, (c) Model 6, (d) Models 4, 5, 7–10, 12, 14. For parameter values in Ω_SSP _both x1∗
 MathType@MTEF@5@5@+=feaafiart1ev1aaatCvAUfKttLearuWrP9MDH5MBPbIqV92AaeXatLxBI9gBaebbnrfifHhDYfgasaacPC6xNi=xH8viVGI8Gi=hEeeu0xXdbba9frFj0xb9qqpG0dXdb9aspeI8k8fiI+fsY=rqGqVepae9pg0db9vqaiVgFr0xfr=xfr=xc9adbaqaaeGacaGaaiaabeqaaeqabiWaaaGcbaGaemiEaG3aa0baaSqaaiabigdaXaqaaiabgEHiQaaaaaa@2F58@ and x2∗
 MathType@MTEF@5@5@+=feaafiart1ev1aaatCvAUfKttLearuWrP9MDH5MBPbIqV92AaeXatLxBI9gBaebbnrfifHhDYfgasaacPC6xNi=xH8viVGI8Gi=hEeeu0xXdbba9frFj0xb9qqpG0dXdb9aspeI8k8fiI+fsY=rqGqVepae9pg0db9vqaiVgFr0xfr=xfr=xc9adbaqaaeGacaGaaiaabeqaaeqabiWaaaGcbaGaemiEaG3aa0baaSqaaiabikdaYaqaaiabgEHiQaaaaaa@2F5A@ are singular variables and approach their threshold values in the step function limit. Then they exhibit threshold robustness for all parameter perturbations which leave the perturbed values inside Ω_SSP_.

Models 1, 2, and 11 have distinctly larger values of *CV*_minmax _than the other models. This behaviour can be explained as a consequence of the shape of the parameter space domain Ω_SSP _in (*μ*_1_, *μ*_2_)-space, where *μ*_*j *_= *α*_*j*_/*γ*_*j*_, *j *= 1, 2. This is the parameter domain for which both x1∗
 MathType@MTEF@5@5@+=feaafiart1ev1aaatCvAUfKttLearuWrP9MDH5MBPbIqV92AaeXatLxBI9gBaebbnrfifHhDYfgasaacPC6xNi=xH8viVGI8Gi=hEeeu0xXdbba9frFj0xb9qqpG0dXdb9aspeI8k8fiI+fsY=rqGqVepae9pg0db9vqaiVgFr0xfr=xfr=xc9adbaqaaeGacaGaaiaabeqaaeqabiWaaaGcbaGaemiEaG3aa0baaSqaaiabigdaXaqaaiabgEHiQaaaaaa@2F58@ and x2∗
 MathType@MTEF@5@5@+=feaafiart1ev1aaatCvAUfKttLearuWrP9MDH5MBPbIqV92AaeXatLxBI9gBaebbnrfifHhDYfgasaacPC6xNi=xH8viVGI8Gi=hEeeu0xXdbba9frFj0xb9qqpG0dXdb9aspeI8k8fiI+fsY=rqGqVepae9pg0db9vqaiVgFr0xfr=xfr=xc9adbaqaaeGacaGaaiaabeqaaeqabiWaaaGcbaGaemiEaG3aa0baaSqaaiabikdaYaqaaiabgEHiQaaaaaa@2F5A@ attain threshold values in the step function limit, in other words, the domain in which there is threshold robustness in both variables. For Models 1, 2, and 11 Ω_SSP _is concave, while for all the other models it is either wedge-shaped or rectangular (Fig. 6). In the concave domains of Models 1, 2, and 11 there is no point giving threshold robustness in both variables for the full parameter perturbation range of 50% up and down (see the Methods section, which also contains the derivation of this result), contrary to all the other models where threshold robustness is obtained for all parameter values sufficiently far from the boundary. With this result in mind it is reasonable to expect a drastically reduced robustness for these three models compared to the rest.

**Figure 7:**
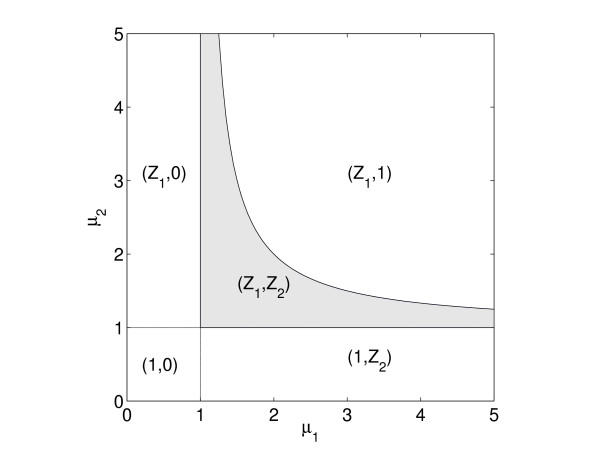
In the case of very steep sigmoids, the (*μ*_1_, *μ*_2_) space of Model 2 is divided into 5 domains, each domain comprising the parameter values giving a particular type of SSP. For example, in the domain denoted (*Z*_1_, 1), *x*_1 _is at its threshold and is singular, thus *Z*_1 _≠ 0, 1, while *x*_2 _is above its threshold, thus *Z*_2 _= 1. Only in the shaded domain are both variables singular and actively regulating. For steep, but not infinitely steep response functions the relations are approximately true.

A comparison of *CV*_1 _and *CV*_2 _for all models show a distinct difference in robustness of *x*_1 _and *x*_2 _for Model 2 and Model 11 (Fig. 5d). We can explain this difference by how the character of the stationary point varies over the parameter space (Fig. 7 for Model 2). One can see that *x*_1 _is singular for all *μ*_2 _> 1 independent of *μ*_1_, while the domain in which *x*_2 _is singular is much smaller and with a strongly narrowing band. In this band, all points are close to the boundary, and robustness in *x*_2 _is very easily lost. Accordingly, the probability of having a perturbed point in parameter space in which the singular state is preserved is much less for *x*_2 _than for *x*_1_, just as seen in Fig. 5. For Model 11 the situation is similar.

To further illustrate the difference between the least robust Models 1, 2, and 11 and the rest of the models, we computed for each realisation the number of perturbations *N *for which the stable point is a SSP with both *x*_1 _and *x*_2 _as active regulators. We consider *x*_*j *_an active regulator if Zj∗
 MathType@MTEF@5@5@+=feaafiart1ev1aaatCvAUfKttLearuWrP9MDH5MBPbIqV92AaeXatLxBI9gBaebbnrfifHhDYfgasaacPC6xNi=xH8viVGI8Gi=hEeeu0xXdbba9frFj0xb9qqpG0dXdb9aspeI8k8fiI+fsY=rqGqVepae9pg0db9vqaiVgFr0xfr=xfr=xc9adbaqaaeGacaGaaiaabeqaaeqabiWaaaGcbaGaemOwaO1aa0baaSqaaiabdQgaQbqaaiabgEHiQaaaaaa@2F89@ lies in the interval ⟨0.05, 0.95⟩. Among all 81 parameter sets the highest observed *N *was 37, 37, and 35 for Models 1, 2, and 11 respectively. For all other models one can always find a parameter set with *N *= 50, i.e. for which all perturbations render a SSP with a high degree of robustness in both x1∗
 MathType@MTEF@5@5@+=feaafiart1ev1aaatCvAUfKttLearuWrP9MDH5MBPbIqV92AaeXatLxBI9gBaebbnrfifHhDYfgasaacPC6xNi=xH8viVGI8Gi=hEeeu0xXdbba9frFj0xb9qqpG0dXdb9aspeI8k8fiI+fsY=rqGqVepae9pg0db9vqaiVgFr0xfr=xfr=xc9adbaqaaeGacaGaaiaabeqaaeqabiWaaaGcbaGaemiEaG3aa0baaSqaaiabigdaXaqaaiabgEHiQaaaaaa@2F58@ and x2∗
 MathType@MTEF@5@5@+=feaafiart1ev1aaatCvAUfKttLearuWrP9MDH5MBPbIqV92AaeXatLxBI9gBaebbnrfifHhDYfgasaacPC6xNi=xH8viVGI8Gi=hEeeu0xXdbba9frFj0xb9qqpG0dXdb9aspeI8k8fiI+fsY=rqGqVepae9pg0db9vqaiVgFr0xfr=xfr=xc9adbaqaaeGacaGaaiaabeqaaeqabiWaaaGcbaGaemiEaG3aa0baaSqaaiabikdaYaqaaiabgEHiQaaaaaa@2F5A@.

## Discussion

A number of different sources of robustness in cellular function and biochemical networks are discussed in the literature (see e.g. [35,36]). Considered as as a systemic property of a developmental or functional unit in an organism, robustness has been explained as a consequence of both negative and positive feedback and several other network properties [30,37,38], as a consequence of network topology and connectivity [39-41] or of modularity or redundancy of the network [42,43]. Conversely, complexity has been seen as a consequence of selection for robustness rather than the other way round [44,45].

Distinguishing the phenomenon of robustness from homeostasis and stability which concern the system's ability to maintain a stable state, Kitano defines robustness as "a property that allows a system to maintain its functions against external perturbations [37,46]." The function maintained by threshold robustness is the gene's or gene product's ability to act as an active regulator of itself or another gene despite parameter perturbations, which can be seen as consequences of external noise.

A variable *x*_*j *_exhibits threshold robustness when it is a singular (also called switching) variable of a SSP, in other words, when the stationary value xj∗
 MathType@MTEF@5@5@+=feaafiart1ev1aaatCvAUfKttLearuWrP9MDH5MBPbIqV92AaeXatLxBI9gBaebbnrfifHhDYfgasaacPC6xNi=xH8viVGI8Gi=hEeeu0xXdbba9frFj0xb9qqpG0dXdb9aspeI8k8fiI+fsY=rqGqVepae9pg0db9vqaiVgFr0xfr=xfr=xc9adbaqaaeGacaGaaiaabeqaaeqabiWaaaGcbaGaemiEaG3aa0baaSqaaiabdQgaQbqaaiabgEHiQaaaaaa@2FC5@ approaches the threshold *θ*_*j *_as the steepness of the associated sigmoid function tends to infinity. In that case the stationary value is locked to the threshold whatever the value of the other parameters (as long they are not perturbed so strongly that *x*_*j *_is no longer a singular variable in the perturbed system). Then the equilibrium value of the response function Zj∗
 MathType@MTEF@5@5@+=feaafiart1ev1aaatCvAUfKttLearuWrP9MDH5MBPbIqV92AaeXatLxBI9gBaebbnrfifHhDYfgasaacPC6xNi=xH8viVGI8Gi=hEeeu0xXdbba9frFj0xb9qqpG0dXdb9aspeI8k8fiI+fsY=rqGqVepae9pg0db9vqaiVgFr0xfr=xfr=xc9adbaqaaeGacaGaaiaabeqaaeqabiWaaaGcbaGaemOwaO1aa0baaSqaaiabdQgaQbqaaiabgEHiQaaaaaa@2F89@ is neither close to 0 nor to 1, these cases corresponding to the gene in question being either constitutively off or constitutively on. We illustrated this by our analysis of Model 2 given above. For all parameter perturbations which maintain the parameter values within the shaded area in Fig. 7, both variables are singular and locked to their threshold, and robustness is preserved. Only if the equilibrium point is perturbed outside the shaded area, one or both xj∗
 MathType@MTEF@5@5@+=feaafiart1ev1aaatCvAUfKttLearuWrP9MDH5MBPbIqV92AaeXatLxBI9gBaebbnrfifHhDYfgasaacPC6xNi=xH8viVGI8Gi=hEeeu0xXdbba9frFj0xb9qqpG0dXdb9aspeI8k8fiI+fsY=rqGqVepae9pg0db9vqaiVgFr0xfr=xfr=xc9adbaqaaeGacaGaaiaabeqaaeqabiWaaaGcbaGaemiEaG3aa0baaSqaaiabdQgaQbqaaiabgEHiQaaaaaa@2FC5@ slip away from threshold onto one of the flatter part of the response curve where corresponding *Z*_*j*_-value approaches 0 or 1 and is rather insensitive to variations in the *x*_*j*_-value. Then the *x*-variable is no longer an active regulator, the previously regulated gene now being either constantly off or constantly on, and its robustness is gone. This analysis pertains to the limiting case of very steep responses functions, but is approximately valid when the response is more gradual.

Thus, threshold robustness of a transcription factor or gene product stems from the fact that it is actively *regulating *itself or other genes in the stable equilibrium state. There is a general theorem stating that the system of equations *F*(*x*) = *g*, where *x *∈ *R*^*n*^, *F *is a differentiable function *F *: *R*^*n *^→ *R*^*n*^, and *F*(*x*_0_) = *g*_0_, can only have a differentiable solution *x *= *G*(*g*), *x*_0 _= *G*(*g*_0_), if there is a feedback loop involving all *n *variables [27,47]. When this is applied to a SSP, it follows that *for the SSP to exist there must be a feedback loop among all the singular variables, mediated by the sigmoidal terms Z_j _*[27]. Thus, at least for the models investigated in the present paper, if there is a sigmoid-mediated feedback loop among a subset of the variables, and there is a SSP in which these variables are singular, and the sigmoids involved are sufficiently steep, the system will exhibit threshold robustness in this SSP for all the singular variables. Mathematically, threshold robustness is not restricted to any particular type of feedback system. Rather, it is a generic feature of GRNs with steep response functions. Due to the generality of the above-mentioned theorem and the concept and properties of SSPs [25,26], we conjecture that threshold robustness is a general property of singular stationary points. Our findings suggest that this feature is generic in a wider sense, not being dependent on response functions being steep, but it becomes weaker as the system approaches Michaelis-Menten conditions. Also, we may conclude that threshold robustness is a systemic property effectuated by the feedback loop between the sigmoidal interaction terms of the variables at threshold.

The fact that an actively regulating variable necessarily is part of a feedback loop explains how it can maintain its regulation ability. Due to the feedback and because the point is stable, a perturbation that shifts a singular variable away from its threshold, will eventually be counteracted. Thus, a singular variable, i.e. a variable with threshold robustness, is actively regulating and also itself being actively regulated. On the other hand, a feedback loop, even between sigmoidal terms, do not necessarily imply that a SSP exists. It may for certain parameter value combinations but not for others. Thus, it would not be fair to say that threshold robustness is a necessary consequence of feedback. Rather, it is a property of a SSP, and the SSP can only exist if there is a sigmoid-mediated feedback loop among its singular variables. René Thomas has suggested the reasonable conjecture that this must be a negative feedback loop [48]. Our results seem to support this conjecture and suggest that threshold robustness is maintained by negative feedback.

In our numerical simulations, we chose both thresholds fixed at *θ*_*j *_= 1. However, this was just a matter of convenience and not a model limitation. Without this choice, all parameter variations would still have been expressed by the same two parameters, but with the modification that *μ*_*j *_= *α*_*j*_/(*γ*_*j*_*θ*_*j*_). Thus, variation in the thresholds would also result in a variation of *μ*_*j*_, the only additional effect being a shift in the position of the SSP as long as the perturbed parameters would not fall outside the domain in parameter space where both variables are singular. The system would still exhibit threshold robustness, but now around the shifted threshold values. For the majority of the models this domain covers the major part of the parameter space (c.f. Fig. 6). For this reason threshold robustness might also be called *adaptive robustness*.

Seen as genotype-phenotype maps the studied gene regulatory networks share some interesting features related to the genetic phenomena hidden genetic variation and neutrality. Hidden (or cryptic) genetic variation is defined as *"standing genetic variation that does not contribute to the normal range of phenotypes observed in a population, but that is available to modify a phenotype that arises after environmental change or the introduction of novel alleles *[49]*."* It is known that negative autoregulation hides variation in the copy numbers of genes [33]. Our results show a more general connection between negative feedback, threshold regulation and hidden genetic variation. In our simulations we fixed a certain amount of parameter variation, corresponding to genetic variation, for a range of different gene regulatory models, and showed that this variation does not result in a corresponding variation in the phenotype. Thus, in a network which exhibits threshold robustness, functional mutations are hidden for phenotypic selection. Our results imply that mutations causing changes in the maximum production rate or the relative decay rate, but keeping the threshold of regulation intact, may have almost no phenotypic signature if the regulatory dose-response relationships are steep enough. Such mutations are neutral in the sense of Wagner's definition: *"A neutral mutation does not change a well defined aspect of a biological system's function in a specific environment and genetic background *[36]*."* It is implicit in this definition that neutral mutations may aquire a phenotypic signature if the system conditions change. Threshold robustness also offers an explanation of how genotypic variation that is hidden under one condition may be released by for instance a mutation causing a functional change in the regulatory machinery. For instance, the hidden genetic variation could be released by a change in the regulatory structure beyond the limit inside which robustness for one or several variables is conserved. That would turn singular components into regular components without threshold robustness and susceptible to parameter variations. Thus, a single key mutation in a regulatory structure may release a substantial amount of hidden, potential variation, as illustrated in a simulation study by Bergman and Siegal [50].

## Conclusion

This paper presents a simulation study of a class of gene regulatory models in which regulation is modelled with sigmoidal response functions combined by operators mimicking Boolean functions. From a mathematical analysis it is known that when the sigmoidal response functions are very steep, the equilibrium values of the regulating agents are locked to the thresholds, thus are very insensitive to perturbations in all parameters except the threshold levels. This implies that they retain their active regulating power despite parameter perturbations. Our simulations show that this threshold robustness is preserved also for more gentle responses, and is qualitatively present even under Michaelis-Menten conditions. Even though the models investigated are simple, there are reasons to believe that they give a phenomenological description of a large number of different system in which the aggregated effects of a series of transcriptional, translational, and post-translational processes as well as protein-protein interactions and metabolic processes can be described by threshold-dominated response functions.

According to Kitano, robustness is an ability to maintain a function under noise-like perturbations. Threshold robustness is the ability of a protein or transcription factor to maintain an active regulation of a gene in homeostasis under external perturbations. The feedback loop that the system necessarily possesses in the homeostatic state, ensures that the robust members of the loop are themselves regulated and kept close to their threshold values.

Some of the 14 models investigated show a much lower degree of robustness than the rest, a fact that we have explained by a specific analysis of the models in which we compared the shape and extension of the parameter space domain in which the robustness property is preserved under parameter perturbations. For the models with lower degree of robustness, this domain is smaller and concavely shaped such that the perturbed values very easily fall outside the robustness domain. We have also seen that one variable may be considerably less robust than the other, despite all response functions being equally steep, and have offered an explanation of this phenomenon.

Threshold robustness may offer increased insight into genetic phenomena such as maintenance and release of genetic variation in evolution, but a closer investigation of these matters was beyond the scope of the present paper.

## Methods

### Model equations and regulatory functions

To simplify the simulations our model system

y˙j=κjRj(Z1,Z2)−λjyj,j=1,...,3,
 MathType@MTEF@5@5@+=feaafiart1ev1aaatCvAUfKttLearuWrP9MDH5MBPbIqV92AaeXatLxBI9gBaebbnrfifHhDYfgasaacPC6xNi=xI8qiVKYPFjYdHaVhbbf9v8qqaqFr0xc9vqFj0dXdbba91qpepeI8k8fiI+fsY=rqGqVepae9pg0db9vqaiVgFr0xfr=xfr=xc9adbaqaaeGacaGaaiaabeqaaeqabiWaaaGcbaqbaeqabeGaaaqaaiqbdMha5zaacaWaaSbaaSqaaiabdQgaQbqabaGccqGH9aqpiiGacqWF6oWAdaWgaaWcbaGaemOAaOgabeaakiabdkfasnaaBaaaleaacqWGQbGAaeqaaOGaeiikaGIaemOwaO1aaSbaaSqaaiabigdaXaqabaGccqGGSaalcqWGAbGwdaWgaaWcbaGaeGOmaidabeaakiabcMcaPiabgkHiTiab=T7aSnaaBaaaleaacqWGQbGAaeqaaOGaemyEaK3aaSbaaSqaaiabdQgaQbqabaGccqGGSaalaeaacqWGQbGAcqGH9aqpcqaIXaqmcqGGSaalcqGGUaGlcqGGUaGlcqGGUaGlcqGGSaalcqaIZaWmcqGGSaalaaaaaa@4F61@

was scaled to give the scaled equations (3)

*x*_*j*_*' *= *α*_*j*_*R*_*j*_(*Z*_1_, *Z*_2_) - *γ*_*j*_*x*_*j*_,   *j *= 1,...,3,

where *α*_3 _∈ ⟨0, 1⟩ and all *γ*_*j *_∈ ⟨0, 1⟩, and *Z*_*j *_= *x*_*j*_^*p*^/(*x*_*j*_^*p *^+ 1). Details are given below.

The regulatory functions *R*_1 _and *R*_2 _were chosen as algebraic equivalents of Boolean functions with two inputs, subject to the requirements ∂*R*_*j*_/∂*Z*_*j *_≤ 0, *j *= 1, 2, and (∂*R*_1_/∂*Z*_2_)(∂*R*_2_/∂*Z*_1_) ≤ 0 to ensure that there is no positive loop in the system and accordingly no multistationarity, and a globally attracting stationary point. Excluding TRUE and FALSE which imply no regulation, and two more functions that do not fulfil the above mono-stationarity conditions, we were left with the 12 regulatory functions listed in Table 1. The algebraic equivalents are computed by the rules given in [25], X_1 _and X_2 _are Boolean variables, X¯
 MathType@MTEF@5@5@+=feaafiart1ev1aaatCvAUfKttLearuWrP9MDH5MBPbIqV92AaeXatLxBI9gBaebbnrfifHhDYfgasaacPC6xNi=xH8viVGI8Gi=hEeeu0xXdbba9frFj0xb9qqpG0dXdb9aspeI8k8fiI+fsY=rqGqVepae9pg0db9vqaiVgFr0xfr=xfr=xc9adbaqaaeGacaGaaiaabeqaaeqabiWaaaGcbaGafeiwaGLbaebaaaa@2D22@_*i *_= NOT X_*i*_; *Z*_1 _and *Z*_2 _are the corresponding sigmoidal functions as defined above. The third function *R*_3_(*Z*_1_, *Z*_2_) = *Z*_1_*Z*_2 _in all cases.

**Table 1:** The 12 Boolean functions used and their algebraic equivalents

	Boolean function	Algebraic equivalent
1	X_1 _AND X_2_	*Z*_1_*Z*_2_
2	X_1 _AND X¯ MathType@MTEF@5@5@+=feaafiart1ev1aaatCvAUfKttLearuWrP9MDH5MBPbIqV92AaeXatLxBI9gBaebbnrfifHhDYfgasaacPC6xNi=xH8viVGI8Gi=hEeeu0xXdbba9frFj0xb9qqpG0dXdb9aspeI8k8fiI+fsY=rqGqVepae9pg0db9vqaiVgFr0xfr=xfr=xc9adbaqaaeGacaGaaiaabeqaaeqabiWaaaGcbaGafeiwaGLbaebaaaa@2D22@_2_	*Z*_1_(1 - *Z*_2_)
3	X¯ MathType@MTEF@5@5@+=feaafiart1ev1aaatCvAUfKttLearuWrP9MDH5MBPbIqV92AaeXatLxBI9gBaebbnrfifHhDYfgasaacPC6xNi=xH8viVGI8Gi=hEeeu0xXdbba9frFj0xb9qqpG0dXdb9aspeI8k8fiI+fsY=rqGqVepae9pg0db9vqaiVgFr0xfr=xfr=xc9adbaqaaeGacaGaaiaabeqaaeqabiWaaaGcbaGafeiwaGLbaebaaaa@2D22@_1 _AND X_2_	(1 - *Z*_1_)*Z*_2_
4	X¯ MathType@MTEF@5@5@+=feaafiart1ev1aaatCvAUfKttLearuWrP9MDH5MBPbIqV92AaeXatLxBI9gBaebbnrfifHhDYfgasaacPC6xNi=xH8viVGI8Gi=hEeeu0xXdbba9frFj0xb9qqpG0dXdb9aspeI8k8fiI+fsY=rqGqVepae9pg0db9vqaiVgFr0xfr=xfr=xc9adbaqaaeGacaGaaiaabeqaaeqabiWaaaGcbaGafeiwaGLbaebaaaa@2D22@_1 _AND X¯ MathType@MTEF@5@5@+=feaafiart1ev1aaatCvAUfKttLearuWrP9MDH5MBPbIqV92AaeXatLxBI9gBaebbnrfifHhDYfgasaacPC6xNi=xH8viVGI8Gi=hEeeu0xXdbba9frFj0xb9qqpG0dXdb9aspeI8k8fiI+fsY=rqGqVepae9pg0db9vqaiVgFr0xfr=xfr=xc9adbaqaaeGacaGaaiaabeqaaeqabiWaaaGcbaGafeiwaGLbaebaaaa@2D22@_2_	(1 - *Z*_1_)(1 - *Z*_2_)
5	X_1_	*Z*_1_
6	X_2_	*Z*_2_
7	X¯ MathType@MTEF@5@5@+=feaafiart1ev1aaatCvAUfKttLearuWrP9MDH5MBPbIqV92AaeXatLxBI9gBaebbnrfifHhDYfgasaacPC6xNi=xH8viVGI8Gi=hEeeu0xXdbba9frFj0xb9qqpG0dXdb9aspeI8k8fiI+fsY=rqGqVepae9pg0db9vqaiVgFr0xfr=xfr=xc9adbaqaaeGacaGaaiaabeqaaeqabiWaaaGcbaGafeiwaGLbaebaaaa@2D22@_1_	1 - *Z*_1_
8	X¯ MathType@MTEF@5@5@+=feaafiart1ev1aaatCvAUfKttLearuWrP9MDH5MBPbIqV92AaeXatLxBI9gBaebbnrfifHhDYfgasaacPC6xNi=xH8viVGI8Gi=hEeeu0xXdbba9frFj0xb9qqpG0dXdb9aspeI8k8fiI+fsY=rqGqVepae9pg0db9vqaiVgFr0xfr=xfr=xc9adbaqaaeGacaGaaiaabeqaaeqabiWaaaGcbaGafeiwaGLbaebaaaa@2D22@_2_	1 - *Z*_2_
9	X_1 _OR X_2_	*Z*_1 _+ *Z*_2 _- *Z*_1_*Z*_2_
10	X¯ MathType@MTEF@5@5@+=feaafiart1ev1aaatCvAUfKttLearuWrP9MDH5MBPbIqV92AaeXatLxBI9gBaebbnrfifHhDYfgasaacPC6xNi=xH8viVGI8Gi=hEeeu0xXdbba9frFj0xb9qqpG0dXdb9aspeI8k8fiI+fsY=rqGqVepae9pg0db9vqaiVgFr0xfr=xfr=xc9adbaqaaeGacaGaaiaabeqaaeqabiWaaaGcbaGafeiwaGLbaebaaaa@2D22@_1 _OR X¯ MathType@MTEF@5@5@+=feaafiart1ev1aaatCvAUfKttLearuWrP9MDH5MBPbIqV92AaeXatLxBI9gBaebbnrfifHhDYfgasaacPC6xNi=xH8viVGI8Gi=hEeeu0xXdbba9frFj0xb9qqpG0dXdb9aspeI8k8fiI+fsY=rqGqVepae9pg0db9vqaiVgFr0xfr=xfr=xc9adbaqaaeGacaGaaiaabeqaaeqabiWaaaGcbaGafeiwaGLbaebaaaa@2D22@_2_	1 - *Z*_1_*Z*_2_
11	X_1 _OR X¯ MathType@MTEF@5@5@+=feaafiart1ev1aaatCvAUfKttLearuWrP9MDH5MBPbIqV92AaeXatLxBI9gBaebbnrfifHhDYfgasaacPC6xNi=xH8viVGI8Gi=hEeeu0xXdbba9frFj0xb9qqpG0dXdb9aspeI8k8fiI+fsY=rqGqVepae9pg0db9vqaiVgFr0xfr=xfr=xc9adbaqaaeGacaGaaiaabeqaaeqabiWaaaGcbaGafeiwaGLbaebaaaa@2D22@_2_	1 - *Z*_2 _+ *Z*_1_*Z*_2_
12	X¯ MathType@MTEF@5@5@+=feaafiart1ev1aaatCvAUfKttLearuWrP9MDH5MBPbIqV92AaeXatLxBI9gBaebbnrfifHhDYfgasaacPC6xNi=xH8viVGI8Gi=hEeeu0xXdbba9frFj0xb9qqpG0dXdb9aspeI8k8fiI+fsY=rqGqVepae9pg0db9vqaiVgFr0xfr=xfr=xc9adbaqaaeGacaGaaiaabeqaaeqabiWaaaGcbaGafeiwaGLbaebaaaa@2D22@_1 _OR X_2_	1 - *Z*_1 _+ *Z*_1_*Z*_2_

Disregarding cases that were equivalent due to the *x*_1_-*x*_2 _symmetry of Eq. (3), we were left with 14 different models (Fig. 3, only *x*_1 _and *x*_2 _are shown). For example, the scaled equation system for Model 6 is

x′1=α1(1−Z1)Z2−γ1x1,x′2=α2(1−Z1)−γ2x2,x′3=α3Z1Z2−γ3x3.
 MathType@MTEF@5@5@+=feaafiart1ev1aaatCvAUfKttLearuWrP9MDH5MBPbIqV92AaeXatLxBI9gBaebbnrfifHhDYfgasaacPC6xNi=xI8qiVKYPFjYdHaVhbbf9v8qqaqFr0xc9vqFj0dXdbba91qpepeI8k8fiI+fsY=rqGqVepae9pg0db9vqaiVgFr0xfr=xfr=xc9adbaqaaeGacaGaaiaabeqaaeqabiWaaaGcbaqbaeWabmqaaaqaaiqbdIha4zaafaWaaSbaaSqaaiabigdaXaqabaGccqGH9aqpiiGacqWFXoqydaWgaaWcbaGaeGymaedabeaakiabcIcaOiabigdaXiabgkHiTiabdQfaAnaaBaaaleaacqaIXaqmaeqaaOGaeiykaKIaemOwaO1aaSbaaSqaaiabikdaYaqabaGccqGHsislcqWFZoWzdaWgaaWcbaGaeGymaedabeaakiabdIha4naaBaaaleaacqaIXaqmaeqaaOGaeiilaWcabaGafmiEaGNbauaadaWgaaWcbaGaeGOmaidabeaakiabg2da9iab=f7aHnaaBaaaleaacqaIYaGmaeqaaOGaeiikaGIaeGymaeJaeyOeI0IaemOwaO1aaSbaaSqaaiabigdaXaqabaGccqGGPaqkcqGHsislcqWFZoWzdaWgaaWcbaGaeGOmaidabeaakiabdIha4naaBaaaleaacqaIYaGmaeqaaOGaeiilaWcabaGafmiEaGNbauaadaWgaaWcbaGaeG4mamdabeaakiabg2da9iab=f7aHnaaBaaaleaacqaIZaWmaeqaaOGaemOwaO1aaSbaaSqaaiabigdaXaqabaGccqWGAbGwdaWgaaWcbaGaeGOmaidabeaakiabgkHiTiab=n7aNnaaBaaaleaacqaIZaWmaeqaaOGaemiEaG3aaSbaaSqaaiabiodaZaqabaGccqGGUaGlaaaaaa@685A@

### Scaling

Here we outline the scaling steps to non-dimensionalise the model equations (4) and standardise the parameter ranges.

1. For *j *= 1, 2, introduce *x*_*j *_= *y*_*j*_/*θ*_*j *_to scale the thresholds for *x*_*j *_to *θ*_*j *_= 1.

2. Assume *λ*_*j *_∈ ⟨0, Λ⟩, *j *= 1, 2, 3, and scale the time *t *by introducing *τ *= Λ*t*.

3. Assume *κ*_3 _∈ ⟨0, *K*⟩ and introduce *x*_3 _= (*K*/*κ*_3_)*y*_3_.

This leads to the scaled equations (3). The new parameters are *α*_*j *_= *κ*_*j*_/(Λ*θ*_*j*_), *j *= 1, 2, *α*_3 _= *κ*_3_/*K*, and *γ*_*j *_= *λ*_*j*_/Λ, *j *= 1, 2, 3, with the properties *α*_3 _∈ ⟨0, 1⟩ and all *γ*_*j *_∈ ⟨0, 1⟩. Below we use *μ*_*j *_= *α*_*j*_/*γ*_*j*_, *j *= 1, 2.

### Sampling

This subsection describes the generation of parameter sets for Eq. (3). The parameters *γ*_1_, *γ*_2_, *γ*_3 _were sampled from independent uniform distributions over ⟨0, 1⟩. Since we were interested in parameter sets that exhibit threshold regulation in the step-function limit, we did not sample the production rates directly but rather worked our way back from the values of the equilibrium values Z1∗
 MathType@MTEF@5@5@+=feaafiart1ev1aaatCvAUfKttLearuWrP9MDH5MBPbIqV92AaeXatLxBI9gBaebbnrfifHhDYfgasaacPC6xNi=xH8viVGI8Gi=hEeeu0xXdbba9frFj0xb9qqpG0dXdb9aspeI8k8fiI+fsY=rqGqVepae9pg0db9vqaiVgFr0xfr=xfr=xc9adbaqaaeGacaGaaiaabeqaaeqabiWaaaGcbaGaemOwaO1aa0baaSqaaiabigdaXaqaaiabgEHiQaaaaaa@2F1C@ and Z2∗
 MathType@MTEF@5@5@+=feaafiart1ev1aaatCvAUfKttLearuWrP9MDH5MBPbIqV92AaeXatLxBI9gBaebbnrfifHhDYfgasaacPC6xNi=xH8viVGI8Gi=hEeeu0xXdbba9frFj0xb9qqpG0dXdb9aspeI8k8fiI+fsY=rqGqVepae9pg0db9vqaiVgFr0xfr=xfr=xc9adbaqaaeGacaGaaiaabeqaaeqabiWaaaGcbaGaemOwaO1aa0baaSqaaiabikdaYaqaaiabgEHiQaaaaaa@2F1E@ in the limit *p *→ ∞. Granted that these lie in ⟨0, 1⟩, the steady state values of *x*_1 _and *x*_2 _are at their thresholds *θ*_*j *_= 1. From the steady state conditions of Eq. (3), the maximal production rates for gene 1 and 2 are then given by

αj=γjRj(Z1∗,Z2∗),j=1,2.
 MathType@MTEF@5@5@+=feaafiart1ev1aaatCvAUfKttLearuWrP9MDH5MBPbIqV92AaeXatLxBI9gBaebbnrfifHhDYfgasaacPC6xNi=xI8qiVKYPFjYdHaVhbbf9v8qqaqFr0xc9vqFj0dXdbba91qpepeI8k8fiI+fsY=rqGqVepae9pg0db9vqaiVgFr0xfr=xfr=xc9adbaqaaeGacaGaaiaabeqaaeqabiWaaaGcbaqbaeqabeGaaaqaaGGaciab=f7aHnaaBaaaleaacqWGQbGAaeqaaOGaeyypa0tcfa4aaSaaaeaacqWFZoWzdaWgaaqaaiabdQgaQbqabaaabaGaemOuai1aaSbaaeaacqWGQbGAaeqaaiabcIcaOiabdQfaAnaaDaaabaGaeGymaedabaGaey4fIOcaaiabcYcaSiabdQfaAnaaDaaabaGaeGOmaidabaGaey4fIOcaaiabcMcaPaaakiabcYcaSaqaaiabdQgaQjabg2da9iabigdaXiabcYcaSiabikdaYiabc6caUaaaaaa@46D9@

For each of the 14 models we generated 81 initial parameter sets *φ*^*k *^= {*α*_1*k*_, *γ*_1*k*_, *α*_2*k*_, *γ*_2*k*_, *α*_3*k*_, *γ*_3*k*_} by taking the equilibrium values (Z1∗
 MathType@MTEF@5@5@+=feaafiart1ev1aaatCvAUfKttLearuWrP9MDH5MBPbIqV92AaeXatLxBI9gBaebbnrfifHhDYfgasaacPC6xNi=xH8viVGI8Gi=hEeeu0xXdbba9frFj0xb9qqpG0dXdb9aspeI8k8fiI+fsY=rqGqVepae9pg0db9vqaiVgFr0xfr=xfr=xc9adbaqaaeGacaGaaiaabeqaaeqabiWaaaGcbaGaemOwaO1aa0baaSqaaiabigdaXaqaaiabgEHiQaaaaaa@2F1C@, Z2∗
 MathType@MTEF@5@5@+=feaafiart1ev1aaatCvAUfKttLearuWrP9MDH5MBPbIqV92AaeXatLxBI9gBaebbnrfifHhDYfgasaacPC6xNi=xH8viVGI8Gi=hEeeu0xXdbba9frFj0xb9qqpG0dXdb9aspeI8k8fiI+fsY=rqGqVepae9pg0db9vqaiVgFr0xfr=xfr=xc9adbaqaaeGacaGaaiaabeqaaeqabiWaaaGcbaGaemOwaO1aa0baaSqaaiabikdaYaqaaiabgEHiQaaaaaa@2F1E@) from the square lattice {0.1, 0.2,...,0.9} × {0.1, 0.2,...,0.9}.

Having drawn a parameter set we introduced variation in the parameters. For each parameter set *φ*^*k *^we generated a set of 50 perturbed parameter values φ˜ℓk
 MathType@MTEF@5@5@+=feaafiart1ev1aaatCvAUfKttLearuWrP9MDH5MBPbIqV92AaeXatLxBI9gBaebbnrfifHhDYfgasaacPC6xNi=xH8viVGI8Gi=hEeeu0xXdbba9frFj0xb9qqpG0dXdb9aspeI8k8fiI+fsY=rqGqVepae9pg0db9vqaiVgFr0xfr=xfr=xc9adbaqaaeGacaGaaiaabeqaaeqabiWaaaGcbaacciGaf8NXdyMbaGaadaqhaaWcbaGaeS4eHWgabaGaem4AaSgaaaaa@305F@, ℓ = 1,...,50 by keeping *γ*_*j *_fixed and sampling *α*_*j *_of all three genes uniformly in the range ⟨*α*_*j*_/2, 3*α*_*j*_/2⟩. This is justified by the fact that the stable state values only depend on the ratios *μ*_*j*_. For each set of perturbed parameter values we used Matlab's odesolver to find the steady state xjℓ∗k
 MathType@MTEF@5@5@+=feaafiart1ev1aaatCvAUfKttLearuWrP9MDH5MBPbIqV92AaeXatLxBI9gBaebbnrfifHhDYfgasaacPC6xNi=xH8viVGI8Gi=hEeeu0xXdbba9frFj0xb9qqpG0dXdb9aspeI8k8fiI+fsY=rqGqVepae9pg0db9vqaiVgFr0xfr=xfr=xc9adbaqaaeGacaGaaiaabeqaaeqabiWaaaGcbaGaemiEaG3aa0baaSqaaiabdQgaQjabloriSbqaaiabgEHiQiabdUgaRbaaaaa@3255@ of the three gene products in Eq. (3).

### Measuring robustness towards pertubation

The robustness properties of the the three gene products in Eq. (3) were evaluated by comparing the coefficient of variation (*CV*) for parameters to the coefficient of variation for the steady state values. For each *k *∈ {1,...,81} the robustness of the steady state values of *x*_*j *_against perturbations in the production rates *α*_*j *_is analysed using the coefficient of variation

CVjk=std({xjℓ∗k}ℓ=1,...,50)mean({xjℓ∗k}ℓ=1,...,50).
 MathType@MTEF@5@5@+=feaafiart1ev1aaatCvAUfKttLearuWrP9MDH5MBPbIqV92AaeXatLxBI9gBaebbnrfifHhDYfgasaacPC6xNi=xI8qiVKYPFjYdHaVhbbf9v8qqaqFr0xc9vqFj0dXdbba91qpepeI8k8fiI+fsY=rqGqVepae9pg0db9vqaiVgFr0xfr=xfr=xc9adbaqaaeGacaGaaiaabeqaaeqabiWaaaGcbaGaem4qamKaemOvay1aa0baaSqaaiabdQgaQbqaaiabdUgaRbaakiabg2da9KqbaoaalaaabaGaee4CamNaeeiDaqNaeeizaqMaeiikaGIaei4EaSNaemiEaG3aa0baaeaacqWGQbGAcqWItecBaeaacqGHxiIkcqWGRbWAaaGaeiyFa03aaSbaaeaacqWItecBcqGH9aqpcqaIXaqmcqGGSaalcqGGUaGlcqGGUaGlcqGGUaGlcqGGSaalcqaI1aqncqaIWaamaeqaaiabcMcaPaqaaiabb2gaTjabbwgaLjabbggaHjabb6gaUjabcIcaOiabcUha7jabdIha4naaDaaabaGaemOAaOMaeS4eHWgabaGaey4fIOIaem4AaSgaaiabc2ha9naaBaaabaGaeS4eHWMaeyypa0JaeGymaeJaeiilaWIaeiOla4IaeiOla4IaeiOla4IaeiilaWIaeGynauJaeGimaadabeaacqGGPaqkaaGaeiOla4caaa@66EF@

As the coefficient of variation is invariant under scaling, the parameter *CV*s will be close to the *CV *of the uniform distribution *U*(0.5, 1.5) which is *CV*_uni _= 1/12
 MathType@MTEF@5@5@+=feaafiart1ev1aaatCvAUfKttLearuWrP9MDH5MBPbIqV92AaeXatLxBI9gBaebbnrfifHhDYfgasaacPC6xNi=xH8viVGI8Gi=hEeeu0xXdbba9frFj0xb9qqpG0dXdb9aspeI8k8fiI+fsY=rqGqVepae9pg0db9vqaiVgFr0xfr=xfr=xc9adbaqaaeGacaGaaiaabeqaaeqabiWaaaGcbaWaaOaaaeaacqaIXaqmcqaIYaGmaSqabaaaaa@2DD0@ ≈ 0.288. Since we wanted to study how the robustness depends on the steepness of the dose-response function, we carried out this procedure for *p *= 1, 2, 5, 10, 20, 50, 100.

### Explaining the differences in robustness among the models

The reason for the reduced robustness of Models 1, 2, and 11 compared to the remaining models can be explained by investigating the shape and size of the robustness domain Ω_SSP _in the (*μ*_1_, *μ*_2_)-plane for which both *x*_1 _and *x*_2 _are active regulators in the stable point in the step function limit. We find that for Models 1, 2, and 11, Ω_SSP _is a narrow, concave stripe, while for the remaining models it is a convex domain (Fig. 6). Below we show how to derive this result for Model 1. The analyses for the rest of the models are similar.

From Fig. 3 it follows that the scaled equations of motion for *x*_1 _and *x*_2 _in Model 1 are

x′1=α1[1−Z1Z2]−γ1x1,x′2=α2[1−Z2+Z1Z2]−γ2x2.
 MathType@MTEF@5@5@+=feaafiart1ev1aaatCvAUfKttLearuWrP9MDH5MBPbIqV92AaeXatLxBI9gBaebbnrfifHhDYfgasaacPC6xNi=xI8qiVKYPFjYdHaVhbbf9v8qqaqFr0xc9vqFj0dXdbba91qpepeI8k8fiI+fsY=rqGqVepae9pg0db9vqaiVgFr0xfr=xfr=xc9adbaqaaeGacaGaaiaabeqaaeqabiWaaaGcbaqbaeWabiqaaaqaaiqbdIha4zaafaWaaSbaaSqaaiabigdaXaqabaGccqGH9aqpiiGacqWFXoqydaWgaaWcbaGaeGymaedabeaakiabcUfaBjabigdaXiabgkHiTiabdQfaAnaaBaaaleaacqaIXaqmaeqaaOGaemOwaO1aaSbaaSqaaiabikdaYaqabaGccqGGDbqxcqGHsislcqWFZoWzdaWgaaWcbaGaeGymaedabeaakiabdIha4naaBaaaleaacqaIXaqmaeqaaOGaeiilaWcabaGafmiEaGNbauaadaWgaaWcbaGaeGOmaidabeaakiabg2da9iab=f7aHnaaBaaaleaacqaIYaGmaeqaaOGaei4waSLaeGymaeJaeyOeI0IaemOwaO1aaSbaaSqaaiabikdaYaqabaGccqGHRaWkcqWGAbGwdaWgaaWcbaGaeGymaedabeaakiabdQfaAnaaBaaaleaacqaIYaGmaeqaaOGaeiyxa0LaeyOeI0Iae83SdC2aaSbaaSqaaiabikdaYaqabaGccqWG4baEdaWgaaWcbaGaeGOmaidabeaakiabc6caUaaaaaa@5D23@

Assume the stationary point is a SSP in which both variables are singular, and that *p *is very large. Then at equilibrium, x1∗
 MathType@MTEF@5@5@+=feaafiart1ev1aaatCvAUfKttLearuWrP9MDH5MBPbIqV92AaeXatLxBI9gBaebbnrfifHhDYfgasaacPC6xNi=xH8viVGI8Gi=hEeeu0xXdbba9frFj0xb9qqpG0dXdb9aspeI8k8fiI+fsY=rqGqVepae9pg0db9vqaiVgFr0xfr=xfr=xc9adbaqaaeGacaGaaiaabeqaaeqabiWaaaGcbaGaemiEaG3aa0baaSqaaiabigdaXaqaaiabgEHiQaaaaaa@2F58@ ≈ *θ*_1 _= 1, x2∗
 MathType@MTEF@5@5@+=feaafiart1ev1aaatCvAUfKttLearuWrP9MDH5MBPbIqV92AaeXatLxBI9gBaebbnrfifHhDYfgasaacPC6xNi=xH8viVGI8Gi=hEeeu0xXdbba9frFj0xb9qqpG0dXdb9aspeI8k8fiI+fsY=rqGqVepae9pg0db9vqaiVgFr0xfr=xfr=xc9adbaqaaeGacaGaaiaabeqaaeqabiWaaaGcbaGaemiEaG3aa0baaSqaaiabikdaYaqaaiabgEHiQaaaaaa@2F5A@ ≈ *θ*_2 _= 1, and 0 <Zi∗
 MathType@MTEF@5@5@+=feaafiart1ev1aaatCvAUfKttLearuWrP9MDH5MBPbIqV92AaeXatLxBI9gBaebbnrfifHhDYfgasaacPC6xNi=xH8viVGI8Gi=hEeeu0xXdbba9frFj0xb9qqpG0dXdb9aspeI8k8fiI+fsY=rqGqVepae9pg0db9vqaiVgFr0xfr=xfr=xc9adbaqaaeGacaGaaiaabeqaaeqabiWaaaGcbaGaemOwaO1aa0baaSqaaiabdMgaPbqaaiabgEHiQaaaaaa@2F87@ < 1. Solving the equilibrium conditions with respect to *μ*_*j*_, *j *= 1, 2 under these assumptions we find that *μ*_*j *_> 1 and

μ1=11−Z1∗Z2∗,μ2=11−Z2∗+Z1∗Z2∗.
 MathType@MTEF@5@5@+=feaafiart1ev1aaatCvAUfKttLearuWrP9MDH5MBPbIqV92AaeXatLxBI9gBaebbnrfifHhDYfgasaacPC6xNi=xI8qiVKYPFjYdHaVhbbf9v8qqaqFr0xc9vqFj0dXdbba91qpepeI8k8fiI+fsY=rqGqVepae9pg0db9vqaiVgFr0xfr=xfr=xc9adbaqaaeGacaGaaiaabeqaaeqabiWaaaGcbaqbaeWabiqaaaqaaGGaciab=X7aTnaaBaaaleaacqaIXaqmaeqaaOGaeyypa0tcfa4aaSaaaeaacqaIXaqmaeaacqaIXaqmcqGHsislcqWGAbGwdaqhaaqaaiabigdaXaqaaiabgEHiQaaacqWGAbGwdaqhaaqaaiabikdaYaqaaiabgEHiQaaaaaGccqGGSaalaeaacqWF8oqBdaWgaaWcbaGaeGOmaidabeaakiabg2da9KqbaoaalaaabaGaeGymaedabaGaeGymaeJaeyOeI0IaemOwaO1aa0baaeaacqaIYaGmaeaacqGHxiIkaaGaey4kaSIaemOwaO1aa0baaeaacqaIXaqmaeaacqGHxiIkaaGaemOwaO1aa0baaeaacqaIYaGmaeaacqGHxiIkaaaaaOGaeiOla4caaaaa@4DC4@

Upon elimination of Z1∗
 MathType@MTEF@5@5@+=feaafiart1ev1aaatCvAUfKttLearuWrP9MDH5MBPbIqV92AaeXatLxBI9gBaebbnrfifHhDYfgasaacPC6xNi=xH8viVGI8Gi=hEeeu0xXdbba9frFj0xb9qqpG0dXdb9aspeI8k8fiI+fsY=rqGqVepae9pg0db9vqaiVgFr0xfr=xfr=xc9adbaqaaeGacaGaaiaabeqaaeqabiWaaaGcbaGaemOwaO1aa0baaSqaaiabigdaXaqaaiabgEHiQaaaaaa@2F1C@ this leads to

μ2=μ1(2−Z2∗)μ1−1.
 MathType@MTEF@5@5@+=feaafiart1ev1aaatCvAUfKttLearuWrP9MDH5MBPbIqV92AaeXatLxBI9gBaebbnrfifHhDYfgasaacPC6xNi=xI8qiVKYPFjYdHaVhbbf9v8qqaqFr0xc9vqFj0dXdbba91qpepeI8k8fiI+fsY=rqGqVepae9pg0db9vqaiVgFr0xfr=xfr=xc9adbaqaaeGacaGaaiaabeqaaeqabiWaaaGcbaacciGae8hVd02aaSbaaSqaaiabikdaYaqabaGccqGH9aqpjuaGdaWcaaqaaiab=X7aTnaaBaaabaGaeGymaedabeaaaeaacqGGOaakcqaIYaGmcqGHsislcqWGAbGwdaqhaaqaaiabikdaYaqaaiabgEHiQaaacqGGPaqkcqWF8oqBdaWgaaqaaiabigdaXaqabaGaeyOeI0IaeGymaedaaOGaeiOla4caaa@3FCA@

Then Ω_SSP _is the domain covered by this family of curves when Z2∗
 MathType@MTEF@5@5@+=feaafiart1ev1aaatCvAUfKttLearuWrP9MDH5MBPbIqV92AaeXatLxBI9gBaebbnrfifHhDYfgasaacPC6xNi=xH8viVGI8Gi=hEeeu0xXdbba9frFj0xb9qqpG0dXdb9aspeI8k8fiI+fsY=rqGqVepae9pg0db9vqaiVgFr0xfr=xfr=xc9adbaqaaeGacaGaaiaabeqaaeqabiWaaaGcbaGaemOwaO1aa0baaSqaaiabikdaYaqaaiabgEHiQaaaaaa@2F1E@ vary between 0 and 1. One easily finds Ω_SSP _to be the domain between the three curves *μ*_1 _= 1, *μ*_2 _= 1, and *μ*_2 _= *μ*_1_/(*μ*_1 _- 1) (Fig. 6a). As both *μ*_1 _and *μ*_2 _are perturbed 50% up and down in our simulations, there is no point in Ω_SSP _in which both perturbed values are bound to stay inside Ω_SSP_.

## Authors' contributions

SWO and EP initiated the work, ABG and EP devised the experimental design, ABG ran all the simulations, carried out the data analysis and designed the figures, all authors contributed to and approved the final manuscript.

## References

[B1] Glass L, Kauffman SA (1972). Co-operative components, spatial localization and oscillatory cellular dynamics. Journal of Theoretical Biology.

[B2] Glass L, Kauffman SA (1973). The logical analysis of continuous, non-linear biochemical control networks. Journal of Theoretical Biology.

[B3] Thomas R (1973). Boolean Formalization of Genetic-Control Circuits. Journal of Theoretical Biology.

[B4] Yuh CH, Bolouri H, Davidson EH (1998). Genomic *cis*-Regulatory Logic: Experimental and Computational Analysis of a Sea Urchin Gene. Science.

[B5] Gardner T, Cantor C, Collins J (2000). Construction of a genetic toggle switch in *Escherichia coli*. Nature.

[B6] Yuh CH, Bolouri H, Davidson EH (2001). *cis*-regulatory logic in the endo16 gene: switching from a specification to a differentiation mode of control. Development.

[B7] Guet CC, Elowitz MB, Hsing WH, Leibler S (2002). Combinatorial synthesis of genetic networks. Science.

[B8] Setty Y, Mayo AE, Surette MG, Alon U (2003). Detailed map of a *cis*-regulatory input function. PNAS.

[B9] Kramer BP, Fischer C, Fussenegger M (2004). BioLogic gates enable logical transcription control in mammalian cells. Biotechnology and Bioengineering.

[B10] Istrail S, Davidson EH (2005). Logic functions of the genomic *cis*-regulatory code. PNAS.

[B11] Mayo AE, Setty Y, Shavit S, Zaslaver A, Alon U (2006). Plasticity of the *cis*-Regulatory Input Function of a Gene. PLoS Biology.

[B12] Goldbeter A, Koshland DE (1981). An Amplified Sensitivity Arising from Covalent Modification in Biological Systems. PNAS.

[B13] Rossi FMV, Kringstein AM, Spicher A, Guicherit OM, Blau HM (2000). Transcriptional control: Rheostat converted to on/off switch. Molecular Cell.

[B14] Biggar SR, Crabtree GR (2001). Cell signaling can direct either binary or graded transcriptional responses. The EMBO Journal.

[B15] Rosenfeld N, Young JW, Alon U, Swain PS, Elowitz MB (2005). Gene Regulation at the Single-Cell Level. Science.

[B16] Kim J, Bates DG, Postlethwaite I, Ma L, Iglesias PA (2006). Robustness analysis of biochemical network models. IEE Proceedings – Systems Biology.

[B17] Wolf DM, Eeckman FH (1998). On the Relationship Between Genomic Regulatory Element Organization and Gene Regulatory Dynamics. Journal of Theoretical Biology.

[B18] Buchler NE, Gerland U, Hwa T (2005). Nonlinear protein degradation and the function of genetic circuits. PNAS.

[B19] Bintu L, Buchler NE, Garcia HG, Gerland U, Hwa T, Kondev J, Phillips R (2005). Transcriptional regulation by the numbers: models. Current Opinion in Genetics & Development.

[B20] Bintu L, Buchler NE, Garcia HG, Gerland U, Hwa T, Kondev J, Kuhlman T, Phillips R (2005). Transcriptional regulation by the numbers: applications. Current Opinion in Genetics & Development.

[B21] Thattai M, van Oudenaarden A (2002). Attenuation of noise in ultrasensitive signaling cascades. Biophysical Journal.

[B22] Hooshangi S, Thiberge S, Weiss R (2005). Ultrasensitivity and noise propagation in a synthetic transcriptional cascade. PNAS.

[B23] Plahte E, Mestl T, Omholt SW (1994). Global Analysis of Steady Points for Systems of Differential-Equations with Sigmoid Interactions. Dynamics and Stability of Systems.

[B24] Mestl T, Plahte E, Omholt S (1995). A mathematical framework for describing and analyzing gene regulatory networks. Journal of Theoretical Biology.

[B25] Plahte E, Mestl T, Omholt SW (1998). A methodological basis for description and analysis of systems with complex switch-like interactions. Journal of Mathematical Biology.

[B26] Plahte E, Kjøglum S (2005). Analysis and generic properties of gene regulatory networks with graded response functions. Physica D: Nonlinear Phenomena.

[B27] Veflingstad SR, Plahte E (2007). Analysis of gene regulatory network models with graded and binary transcriptional responses. Biosystems.

[B28] Cherry J, Adler F (2000). How to make a biological switch. Journal of Theoretical Biology.

[B29] Brazhnik P, de la Fuente A, Mendes P (2002). Gene networks: how to put the function in genomics. Trends in Biotechnology.

[B30] Becskei A, Serrano L (2000). Engineering stability in gene networks by autoregulation. Nature.

[B31] Rosenfeld N, Elowitz MB, Alon U (2002). Negative autoregulation speeds the response times of transcription networks. Journal of Molecular Biology.

[B32] de Jong H (2002). Modeling and Simulation of Genetic Regulatory Systems: A Literature Review. Journal of Computational Biology.

[B33] Thomas R, D'Ari R (1990). Biological Feedback.

[B34] Alon U (2007). An Introduction to Systems Biology Design Principles of Biological Circuits.

[B35] Stelling J, Sauer U, Szallasi Z, Doyle FJ, Doyle J (2004). Robustness of Cellular Functions. Cell.

[B36] Wagner A (2005). Robustness and Evolvability in Living Systems.

[B37] Kitano H (2004). Biological robustness. Nature Reviews Genetics.

[B38] Venkatesh KV, Bhartiya S, Ruhela A (2004). Multiple feedback loops are key to a robust dynamic performance of tryptophan regulation in *Escherichia coli*. FEBS Letters.

[B39] Bhartiya S, Chaudhary N, Venkatesh KV, Doyle FJ (2006). Multiple feedback loop design in the tryptophan regulatory network of *Escherichia coli *suggests a paradigm for robust regulation of processes in series.. Journal of the Royal Society Interface.

[B40] Ma W, Lai L, Ouyang Q, Tang C (2006). Robustness and modular design of the *Drosophila *segment polarity network. Molecular Systems Biology.

[B41] Siegal ML, Bergman A (2002). Waddington's canalization revisited: Developmental stability and evolution. PNAS.

[B42] von Dassow G, Meir E, Munro E, Odell G (2000). The segment polarity network is a robust development module. Nature.

[B43] Albert R, Jeong H, Barabasi AL (2000). Error and attack tolerance of complex networks. Nature.

[B44] Hartwell LH, Hopfield JJ, Leibler S, Murray AW (1999). From molecular to modular cell biology. Nature.

[B45] Lauffenburger DA (2000). Cell signaling pathways as control modules: Complexity for simplicity?. PNAS.

[B46] Kitano H (2007). Towards a theory of biological robustness. Mol Syst Biol.

[B47] Thomas R, Kaufman M (2001). Multistationarity, the basis of cell differentiation and memory. I. Structural conditions of multistationarity and other nontrivial behavior. Chaos.

[B48] Thomas R (2006). Circular causality. IEE Proceedings Systems Biology.

[B49] Gibson G, Dworkin I (2004). Uncovering cryptic genetic variation. Nat Rev Genet.

[B50] Bergman A, Siegal ML (2003). Evolutionary capacitance as a general feature of complex gene networks. Nature.

